# Validation of ^64^Cu-ATSM damaging DNA via high-LET Auger electron emission

**DOI:** 10.1093/jrr/rrv042

**Published:** 2015-08-06

**Authors:** Dayton D. McMillan, Junko Maeda, Justin J. Bell, Matthew D. Genet, Garrett Phoonswadi, Kelly A. Mann, Susan L. Kraft, Hisashi Kitamura, Akira Fujimori, Yukie Yoshii, Takako Furukawa, Yasuhisa Fujibayashi, Takamitsu A. Kato

**Affiliations:** 1 Department of Environmental and Radiological Health Sciences, Colorado State University, Fort Collins, Colorado 80523, USA; 2 Research, Development and Support Center, National Institute of Radiological Sciences, 4-9-1 Anagawa, Inage, Chiba 263-8555, Japan; 3 Research Center for Radiation Protection, National Institute of Radiological Sciences, 4-9-1 Anagawa, Inage, Chiba 263-8555, Japan; 4 Molecular Imaging Center, National Institute of Radiological Sciences, 4-9-1 Anagawa, Inage, Chiba 263-8555, Japan

**Keywords:** auger electron, high LET, ^64^Cu-ATSM, DNA double strand break, CHO

## Abstract

Radioactive copper (II) (diacetyl-bis N4-methylthiosemicarbazone) (Cu-ATSM) isotopes were originally developed for the imaging of hypoxia in tumors. Because the decay of a ^64^Cu atom is emitting not only positrons but also Auger electrons, this radionuclide has great potential as a theranostic agent. However, the success of ^64^Cu-ATSM internal radiation therapy would depend on the contribution of Auger electrons to tumor cell killing. Therefore, we designed a cell culture system to define the contributions to cell death from Auger electrons to support or refute our hypothesis that the majority of cell death from ^64^Cu-ATSM is a result of high-LET Auger electrons and not positrons or other low-LET radiation. Chinese hamster ovary (CHO) wild type and DNA repair–deficient xrs5 cells were exposed to ^64^Cu-ATSM during hypoxic conditions. Surviving fractions were compared with those surviving gamma-radiation, low-LET hadron radiation, and high-LET heavy ion exposure. The ratio of the D_10_ values (doses required to achieve 10% cell survival) between CHO wild type and xrs5 cells suggested that ^64^Cu-ATSM toxicity is similar to that of high-LET Carbon ion radiation (70 keV/μm). γH2AX foci assays confirmed DNA double-strand breaks and cluster damage by high-LET Auger electrons from ^64^Cu decay, and complex types of chromosomal aberrations typical of high-LET radiation were observed after ^64^Cu-ATSM exposure. The majority of cell death was caused by high-LET radiation. This work provides strong evidence that ^64^Cu-ATSM damages DNA via high-LET Auger electrons, supporting further study and consideration of ^64^Cu-ATSM as a cancer treatment modality for hypoxic tumors.

## INTRODUCTION

Radiolabeled copper (II) (diacetyl-bis N4-methylthiosemicarbazone) (Cu-ATSM) was originally designed as a positron emission tomography (PET) agent [1]. Later, researchers found Cu-ATSM preferentially accumulates in hypoxic tumor regions, enabling non-invasive identification and quantification of hypoxic tissue [2] and providing a clinically appealing method for assessing a tumor's oxygenation state. Hypoxic tumors are resistant to conventional radiation therapy and chemotherapy due to less oxygen tension and poor drug delivery. Therefore, the tumor's hypoxic state has been known as a useful prognostic factor for many years. Recently, it has been discovered that Cu-ATSM-positive but fluorodeoxyglucose (FDG)-negative tumor regions are rich in cancer stem cells [3]. Cancer stem cells are a subpopulation within tumors that may be involved in tumor progression and resistance to cancer treatments, including radiation therapy and chemotherapy. Therefore, radiolabeled Cu-ATSM is a promising agent for targeting the regions related to therapeutic resistance.

The design of Cu-ATSM with a variety of radioactive Cu nuclides allows for the development of a new Cu-ATSM as a theranostic: namely ^64^Cu-ATSM for internal radiation therapy with simultaneous monitoring of the radiation dose. Use of ^64^Cu-ATSM for internal radiation therapy with simultaneous monitoring of the radiation dose is attractive based on a number of critical factors [4]. It decays by emission of β^+^ (0.656 MeV, 19%), β^–^ (0.573 MeV, 40%), γ (1.346 MeV, 0.5%) and electron capture (41%), with a half-life of 12.7 h [5]. Electron capture results in cascades of high-LET Auger electrons, which can overcome a low oxygen enhancement ratio (OER) in hypoxic tumors [6] and kill tumor cells efficiently with higher relative biological effectiveness (RBE) [7] than low-LET radiation. There are several indications that high-LET radiation can efficiently kill radioresistant cells, including cancer stem cells [8, 9]. Auger electrons from ^64^Cu decay have an average of 2 keV of energy, ∼126 nm range in tissue [10], and are considered high-LET radiation. Therefore, when a ^64^Cu atom is taken into a hypoxic cell and decays within range of tumor cell DNA, the emitted Auger electrons can kill tumor cells. If the high-LET Auger electrons are the primary cause of cell death, ^64^Cu-ATSM would be ideal to not only target the radioresistant hypoxic tumor region but also the radioresistant cancer stem cells. Although previous studies have clearly shown the cytotoxicity of ^64^Cu-ATSM in tumor cells from DNA damage [11], there is no direct evidence that Auger electrons are the primary cause of cell death after ^64^Cu-ATSM treatment.

This study evaluated the hypothesis that the majority of cell death from ^64^Cu-ATSM exposure is from high-LET Auger electrons. To characterize cell death from high-LET radiation damage, DNA repair–proficient and –deficient Chinese hamster ovary (CHO) cell lines were used in this study. We exposed those cells to ^64^Cu-ATSM and other types of ionizing radiation with a variety of LET qualities to determine the similarity of the cell death trends. DNA double-strand breaks and chromosomal aberrations after ^64^Cu-ATSM exposure were analyzed to determine the characteristics of DNA damage produced by radiation originating from ^64^Cu.

## MATERIALS AND METHODS

### ^64^Cu-ATSM preparation

^64^Cu, generated by a ^64^Ni(p,n)^64^Cu reaction [12], was purchased from the University of Wisconsin–Madison Cyclotron Research Group (Madison, WI). The preparation of ^64^Cu-ATSM was performed as previously reported [13, 14]. The radiolabeling efficiency of ^64^Cu-ATSM was determined using silica gel thin-layer chromatography with ethyl acetate as the mobile phase [15]. The radiolabeling efficiency was an average of 93.1%, with a standard deviation of 4.7 (*n* = 8).

### Cell culture

Wild-type CHO cells (CHO10B2) and the non-homologous end-joining (NHEJ) repair-deficient xrs5 cells (*ku80* deficient) were graciously supplied by Dr Joel Bedford (Colorado State University, Fort Collins, CO) [16]. Cell cultures were maintained in Eagle's Minimal Essential Medium Alpha (MEM-α) (Gibco, Indianapolis, IN) augmented with 10% heat inactivated fetal bovine serum (FBS, Sigma, St Louis, MO), 1% Penicillin and Streptomycin anti-microbial and 0.1% Fungizone antimycotic (Gibco). Cells were maintained in 5% CO_2_ at 37°C in a humidified incubator. Cell doubling time is ∼12.3 h for CHO wild type and ∼15.0 h for xrs5 cells. Exponential growth cells were used for ^64^Cu-ATSM uptake measurement and all cell survival experiments. G1 cell synchronization was carried out for ***γ***H2AX foci analysis and chromosomal aberration assays. G1 cell synchronization was confirmed with a FACSCalibur flow cytometer (BD Biosciences, Franklin Lakes, NJ).

### Hypoxic treatments and ^64^Cu-ATSM treatment

Cells to be incubated with ^64^Cu-ATSM in hypoxic conditions were pretreated in anoxic conditions 1 h prior to the addition of ^64^Cu-ATSM. Anoxic conditions were maintained in a manner similar to previous studies [11]. Briefly, cell cultures were kept in a 37°C incubator with a continuous flow of 100% nitrogen gas for 1 h [17]. Hypoxia was achieved using the AnaeroPack system (Mitsubishi Gas Chemical, Tokyo, Japan) [18]. The cell cultures were placed into an airtight container with AnaeroPack oxygen-absorbing and CO_2_-generating agents to reduce the O_2_ concentration to <1%. The cell cultures were treated with^64^Cu-ATSM in this hypoxic chamber for 3 h at 37°C.

### ^64^Cu-ATSM uptake

Cells were plated at 200 000 cells per 60 mm cell culture dish and incubated overnight. A total of 185 000 Bq (5 μCi) of ^64^Cu-ATSM was then added to each dish. After incubation for 3 h, cells were washed with PBS and trypsinized. Media, PBS and cells with trypsin were individually retained and mixed with Ultima Gold liquid scintillation cocktail (PerkinElmer, Waltham, MA) to analyze radioactivity in each phase. Vials were shaken and counted on a Beckman LS-5801 liquid scintillation counter (Beckman Coulter, Brea, CA) using the full channel window. Quench differences between the vial compositions were accounted for to determine the relative activity in the vials containing media, PBS and trypsinized cells. Activity present in trypsinized cells was assumed to solely result from cellular incorporation of ^64^Cu.

### External irradiation

Exponentially growing cells were exposed to radiation at room temperature. Gamma-ray irradiations (LET 0.3 keV/µm) were carried out at a dose rate of 2.5 Gy/min using a Model Mark I-68A nominal 222TBq (6,000 Ci) ^137^Cesium sealed source (J.L. Shepherd, Carlsbad, CA). Hadron radiation experiments were carried out at the National Institute of Radiological Sciences (NIRS) in Chiba, Japan [19]. Protons were accelerated to 70 MeV (LET 1.1 keV/µm) using the NIRS-930 cyclotron at NIRS [20]. Carbon-ions and iron-ions were accelerated to 290 MeV/n and 500 MeV/n, respectively, using the heavy ion medical accelerator (HIMAC) at NIRS. The LET of the entrance region for monoenergetic carbon ions and monoenergetic iron ions were 13 and 200 keV/μm, respectively. Monoenergetic carbon ions with a LET of 70 keV/μm were obtained by Lucite attenuation. The dose-averaged LET of the carbon ions at the middle of the 6-cm spread-out Bragg peak (SOBP) is ∼50 keV/µm at a distance of 119 mm from the entrance [21]. Dose rates for carbon-ions, iron-ions and protons were set at 3 Gy/min. All irradiation was carried out at room temperature. LET values of the various radiation types are summarized in Table 1.

**Table 1.** Ratios of D_10_ doses for CHO wild-type and xrs5 cells exposed to various radiations

Radiation	LET keV/μm	(A) D_10_ of CHO10B2	(B) D_10_ of xrs5	Ratio (A)/(B)
Gamma rays	0.3	6.37 Gy	1.18 Gy	5.40
Proton Mono	1.1	5.31Gy	1.16 Gy	4.58
Carbon Mono	13	3.79 Gy	0.91 Gy	4.16
Carbon SOBP	50	3.16 Gy	1.05 Gy	3.01
Carbon Mono	70	2.49 Gy	0.94 Gy	2.65
Iron Mono	200	1.89 Gy	1.00 Gy	1.89
^64^Cu-ATSM	NA	0.97 Bq/cell	0.40 Bq/cell	2.43

### Colony formation assay

For the external irradiation, exponentially growing cells were exposed to the radiation sources described above. Cells were then trypsinized and immediately plated in dishes at an appropriate cell density for colony forming. For the internal irradiation by ^64^Cu-ATSM, single cells were plated onto culture flasks and allowed to adhere for 2 h. After various doses of ^64^Cu-ATSM incubation, all cells were incubated under normoxic conditions for colony formation. Colonies were fixed and stained 7–10 days later using 100% ethanol followed by 0.1% crystal violet. Macroscopic colonies containing >50 cells were marked as survivors [22]. Each experiment was performed at least three times. Cell-survival curves were drawn from cell-surviving fractions in GraphPad Prism 6 (GraphPad, La Jolla, CA) using a linear quadratic regression model. D_10_ values (radiation doses required to achieve 10% cell survival) were obtained from the cell survival curves using a linear quadratic regression model. The ratios of D_10_ doses between CHO wild type and xrs5 were used as a relative metric for the LET of the radiation.

### γH2AX foci formation assay

Cells were synchronized on chamber slides in the G1 phase by mitotic shake off and 2 h of incubation [23]. Following ^64^Cu-ATSM incubation for 3 h, cells were fixed in 4% paraformaldehyde and permeabilized for 5 min in 0.2% Triton X-100 (Sigma, St Louis, MO) in PBS, and blocked overnight at 4°C in 10% goat serum solution. Immunostaining was carried out with anti-γH2AX mouse monoclonal antibody (Millipore, Billerica, MA) and Alexa Fluor488-conjugated goat anti-mouse IgG antibody (Invitrogen, Grand Island, NY). Cells were mounted with ProLong Gold Antifade Reagent with DAPI (Invitrogen). Fluorescence images were captured using a Zeiss Axioskop motorized z-stage fluorescent microscope (Zeiss, Jena, Germany) equipped with CoolSNAP HQ2 (Photometrics, Tucson AZ). One-micron-thick z-stack images were obtained by Metamorph software (Molecular Devices, Sunnyvale, CA). Three independent experiments were performed. Manual counting was performed for foci analysis.

### Chromosomal aberration assay

Cells were synchronized into the G1 phase by mitotic shake off and 2 h of incubation. After ^64^Cu-ATSM was added, cells were incubated for 3 h under hypoxic conditions, then 0.1 μg/ml Colcemid (KaryoMAX® Colcemid™ Solution in PBS, Gibco) was added and incubated for 12–16 h under normoxic conditions. Since we observed a wide variety in a degree of chromosome aberrations after ^64^Cu-ATSM exposure, we assumed faster entry of less damaged cells and slower entry of heavily damaged cells to mitosis. This prolonged Colcemid treatment was aimed to collect a maximum population in metaphase cells. Harvested cells were treated with hypotonic solution (75 mM KCl) for 20 min at 37°C and fixed with methanol:acetic acid (3:1) solution three times before being dropped onto slides. Samples were stained with 5% (v/v) Giemsa solution in Gurr's buffer (Gibco). At least 50 metaphase cells were scored in at least two separate experiments. Chromosomal aberrations were quantified and classified as various chromosome and chromatid type aberrations.

### Statistical methods

Analysis of variance was used to determine statistical significance with GraphPad Prism 6. Standard errors of the means for data were calculated and were depicted in each figure.

## RESULTS

### ^64^Cu-ATSM uptake

**Fig. 1.**
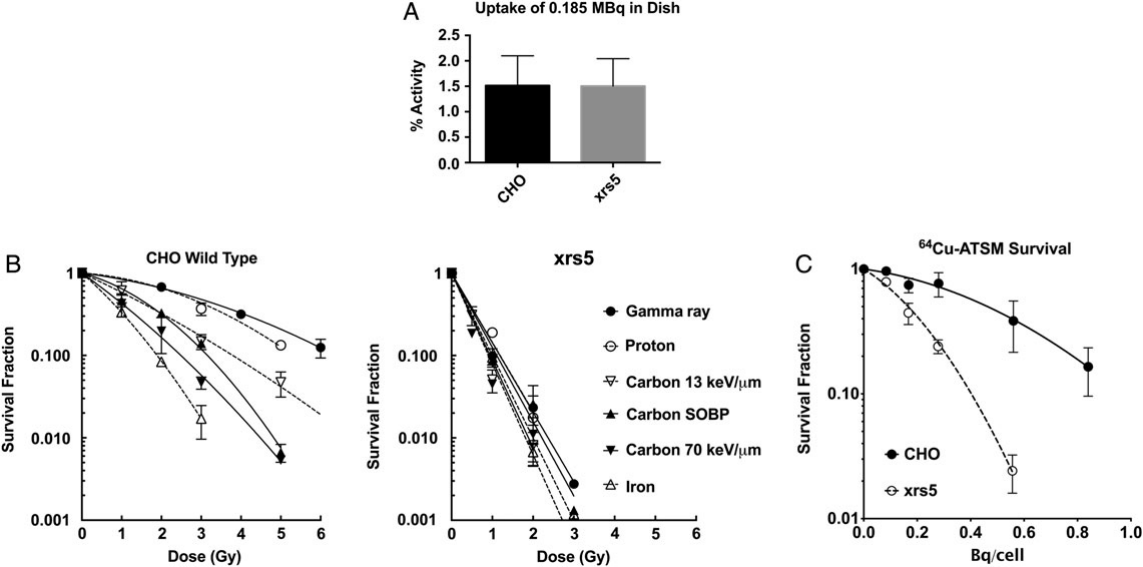
^64^Cu-ATSM uptake and survival curves. (**A**) ^64^Cu-ATSM uptake in CHO wild-type and xrs5 cells. (**B**) Survival curves of CHO wild-type and xrs5 cells exposed to radiation of varying LETs. (**C**) Cell-survival curves of CHO wild-type and xrs5 cells exposed to ^64^Cu-ATSM and incubated in hypoxia. Error bars indicate standard errors of the mean of at least three independent experiments.

The amount of cellular uptake of ^64^Cu was compared at 0.185 MBq in P60 dishes under hypoxic conditions (Fig. 1A). Both DNA repair–proficient CHO wild type and –deficient xrs5 cells showed an uptake of ∼1.4% of total activity. There were no significant differences in uptake between cell lines. Therefore, mutation of Ku80 in xrs5 did not affect cellular uptake of ^64^Cu for CHO cells.

### Colony formation assay

Fig. 1B shows the cell survival curves of CHO wild-type and xrs5 cells after various types of ionizing radiation exposure. CHO wild-type cells showed higher radiosensitivity to higher-LET radiation such as carbon ion LET 70 keV/μm and iron ion LET 200 keV/μm. xrs5 cells were more sensitive than CHO wild-type cells for all types of ionizing radiation. Radiosensitivity of xrs5 was dependent on absorbed dose but not LET.

Figure 1C shows the cell-survival curves of CHO wild-type and xrs5 cells exposed to ^64^Cu-ATSM. CHO wild-type cells were more resistant to ^64^Cu-ATSM than xrs5 cells. The cell-survival curves against ^64^Cu-ATSM exposure fit well with the linear quadratic regression curves.

D_10_ values were obtained from linear quadratic regression curves (Table 1). D_10_ values for CHO wild-type cells ranged from 1.89 to 6.37 Gy, while values for xrs5 deviated very little from 1.00 to 1.18 Gy (iron and gamma irradiation, respectively) (Table 1). Ratios of D_10_ doses (CHO WT/xrs5) were evaluated and used as a relative metric for the LET of the radiation with lower ratios, closer to the high-LET carbon ions or iron ions, indicating higher LET. Survival curves for cells exposed to ^64^Cu-ATSM showed a D_10_ ratio of 2.43, just smaller than that of the carbon ion LET 70 keV/μm (ratio = 2.65).

### γH2AX foci formation assay

**Fig. 2.**
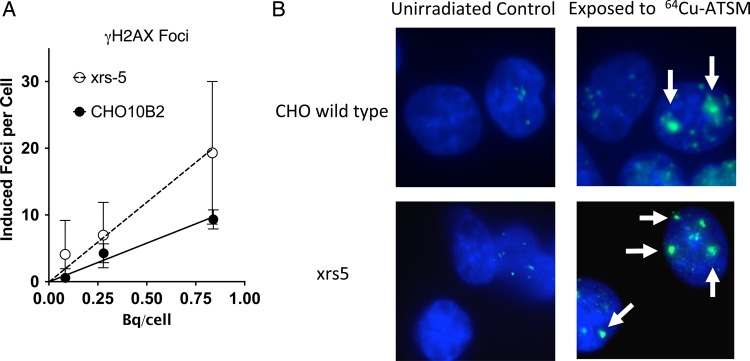
γH2AX foci formation after ^64^Cu-ATSM exposures. (**A**) Dose response of γH2AX foci formation after ^64^Cu-ATSM exposures. Foci response of cells incubated with varied activities normalized to background. Data points are the mean of at least three experiments. Error bars represent the standard error of the mean. (**B**) Examples of γH2AX foci formation in CHO wild-type and xrs5 cells without irradiation and after 0.84 and 0.83 Bq/cell of ^64^Cu-ATSM exposure. Arrows indicate cluster foci.

Cells exposed to ^64^Cu-ATSM under hypoxic conditions showed the formation of γH2AX foci, and it was ^64^Cu dose dependent (Fig. 2A). This directly supports the hypothesis that ^64^Cu-ATSM treatments can produce DNA double-strand breaks. The number of induced foci per cell was twice as much in xrs5 compared with that in CHO wild-type cells. For every Bq per cell of uptake, the foci count increased by 11.6 for CHO wild-type cells and by 23.9 for xrs5 cells with the 3 h incubation of ^64^Cu-ATSM. We also observed several clusters of foci in both CHO wild-type and xrs5 cells after ^64^Cu-ATSM exposures (Fig. 2B). The difference in the number of foci between CHO wild-type and xrs5 cells may result from the differences in repair capacity during the 3 h of exposure time to ^64^Cu-ATSM.

### Chromosomal aberrations assay

In the chromosomal aberrations assay, we observed both chromatid- and chromosome-type aberrations in CHO wild-type cells after ^64^Cu-ATSM treatment, and only chromatid-type aberrations in xrs5 cells (Table 2). Cells were initially synchronized into G1 and exposed to ^64^Cu-ATSM. When DNA double-strand breaks are produced in G1, chromosome-type aberrations are formed. In S and G2, chromatid-type aberrations are formed instead. ^64^Cu decays during cell cycle progression and damages DNA in G1, S and G2 phases before cells reach mitosis. Therefore, both chromatid- and chromosome-type aberrations were observed in our experimental conditions.

**Table 2.** Chromosomal aberrations of CHO10B2 and xrs5 cells exposed to ^64^Cu-ATSM

Dose (Bq/cell)	Total number of cell scored	Total chromosomal aberrations per cell	Total chromatid type per cell	Chromatid breaks per cell	Chromatid exchange per cell	Total chromosome type per cell	Interstitial & terminal deletion per cell	Dicentric & ring per cell
**CHO10B2**								
0	197	0.036	0.015	0.015	0	0.02	0.02	0
0.084	200	0.105	0.050	0.05	0	0.055	0.04	0.015
0.28	188	0.500	0.388	0.272	0.117	0.112	0.09	0.021
0.84	208	0.707	0.591	0.438	0.154	0.115	0.072	0.044
**xrs5**								
0	139	0.309	0.266	0.244	0.022	0.043	0.022	0.021
0.083	145	1.694	1.660	1.34	0.319	0.035	0.007	0.028

CHO wild-type and xrs5 cells showed more chromatid-type aberrations than chromosome-type aberrations. Dose-dependent increases of both chromatid- and chromosome-type aberrations, up to 0.84 Bq per cell of ^64^Cu, were observed in CHO wild-type cells. On the other hand, xrs5 cells showed more noticeable increases in chromatid breaks at 0.083 Bq per cell of ^64^Cu. The background yields of chromosomal aberrations in xrs5 cells were relatively higher than previously reported values [24]. It is possible that radiosensitive xrs5 cells were affected by gamma rays from ^64^Cu decay in adjacent flasks in the incubator.

**Fig. 3.**
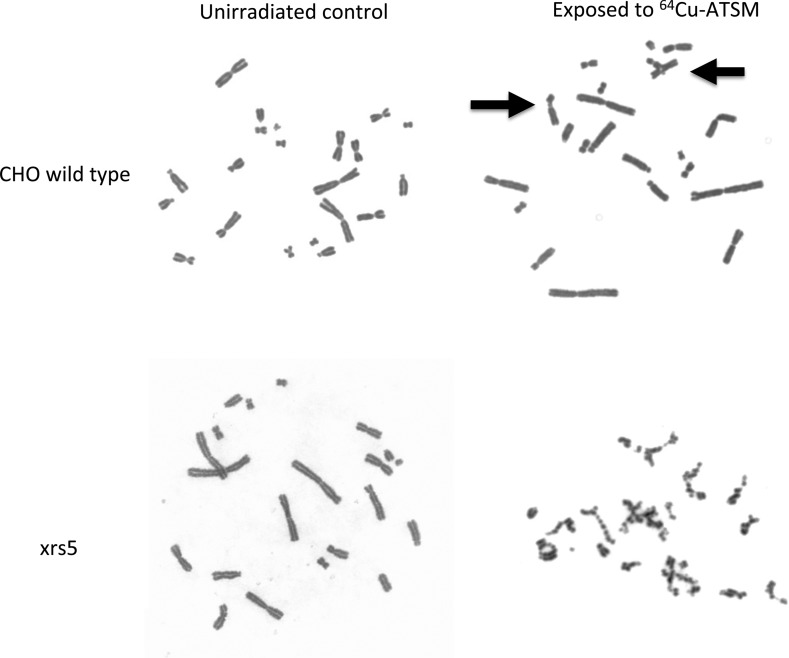
Representative chromosome spreads for CHO wild-type and xrs5 cells in control group and exposed to 0.84 Bq/cell and 0.83 Bq/cell of ^64^Cu-ATSM, respectively. Arrows indicate chromatid-type aberrations in CHO wild-type cells after exposure to ^64^Cu-ATSM. On the other hand, damage in xrs5 was extensive enough to prohibit evaluation of definitive chromosomal aberrations after exposure to 0.83 Bq/cell of ^64^Cu-ATSM.

We could not determine the yield of chromosomal aberrations above 0.27 Bq per cell of ^64^Cu for xrs5 cells because of the severity of the chromatid-type aberrations (Fig. 3). The severe damage to chromosomes observed in the two highest activity experiments for xrs5 cells still yielded metaphase spreads, but the damages were extensive enough to prohibit chromosome aberration qualification and quantification.

Chromatid-type aberrations were dominant in both cell lines for ^64^Cu-ATSM-induced chromosomal aberrations in our experimental conditions. The half-life of ^64^Cu is 12.7 h, and the cells were constantly exposed to radiation from ^64^Cu during the 3-h incubation period. Heavily damaged cells with chromosome-type aberrations produced in G1 stopped the cell cycle by checkpoint activation and did not reach metaphase.

## DISCUSSION

The object of this study was to evaluate the role of Auger electron–induced cell killing as a mechanism of cell death after ^64^Cu-ATSM treatment. The complex pattern of decay modes of ^64^Cu, including Auger electron emissions, led to difficulties for physics studies. The largest problem was the estimation of absorbed dose from ^64^Cu. Monte Carlo simulation of decay of ^64^Cu and information about ^64^Cu distribution within the cells is required in order to estimate an accurate dose to the nuclei. We used the activity in the cell as a general measure of activity to estimate the radiation exposure to cells (Fig. 1A). The observed uptake in both of the cell lines was lower than in other reported tumor cell lines [11]. This may result from the relatively normal metabolic activity in the CHO cell lines.

Even with this relatively low uptake of ^64^Cu into the cells, cell-survival curves for CHO wild-type cells were comparable with the previous report of cell killing [11]. Radiosensitive xrs5 cells showed higher sensitivity to ^64^Cu-ATSM than CHO wild-type cells. Because there were no statistical differences in uptake of ^64^Cu activities in both the cell lines (Fig. 1A), the higher sensitivity to ^64^Cu-ATSM in xrs5 cells can be contributed to the DNA repair deficiency in xrs5 cells.

D_10_ values of repair-proficient cells decrease with LET because there is more cell death in high-LET radiation. On the other hand, it is known that NHEJ repair–deficient mutants show a smaller decrease in D_10_ values with LET. Especially, the D_10_ values of xrs5 were independent of LET [24, 25]. Late S phase of CHO wild-type cells are radioresistant. On the other hand, xrs5 cells do not have cell cycle–dependent cellular radiosensitivity [26]. NHEJ-deficient xrs5 and V3 cells are more sensitive to ionizing radiation than homologous recombination repair–deficient 51D1 and irs1SF cells [27]. This suggests that Ku80 proteins and the NHEJ repair pathway have a central role in radiation-induced damage repair. We compared the ratios between D_10_ values of wild-type and xrs5 cells to estimate the mean LET of lethal events associated with radiation from ^64^Cu-ATSM. The exposure to radiation from ^64^Cu-ATSM can be classified as low-dose-rate radiation because ^64^Cu's half-life is ∼12 h. However, the dose rate effect is known to be smaller in high LET [28]. The ratio of D_10_ values after ^64^Cu-ATSM exposure was in the range of high-LET radiation and were similar to those with 70 keV/μm carbon ions. Therefore, the dose rate effect may be a minor issue for ^64^Cu-ATSM exposure. This D_10_ ratio comparison supports the hypothesis that ^64^Cu-ATSM primarily kills cells with high-LET Auger electrons. However, one concern of our analysis may be that we conducted external irradiation under normoxic conditions. One report showed hypoxic conditions changed D_10_ values of CHO-K1 cells for X-rays from 3.8 Gy to 10.5 Gy and for carbon ions with LET of 80 keV/μm from 1.8 Gy to 3.3 Gy, normoxic and hypoxic, respectively [29]. In future studies, internal and external irradiation methods will be conducted under identical conditions.

Quantitative results of the γH2AX experiments showed a linear increase in foci formation for both CHO cell lines, as in our previous report with X-rays [30]. The NHEJ mutant xrs5 cells showed a more rapid increase in the γH2AX number compared with CHO wild-type cells. A high degree of variability was observed in the foci analysis due to what appeared to be occasional highly damaged cells and intense cluster foci (Fig. 2A). Since cells were synchronized into G1 phase after mitotic shake off and the additional 3 h treatment time with ^64^Cu-ATSM in hypoxia, there was not enough time for cells to enter S phase. We could exclude the possibility of any replication-dependent foci formation. Therefore, this is thought to be due to heterogeneous ^64^Cu-ATSM uptake in the cell population or to the difficulty of manual counting of the intense cluster foci. In addition to the quantitative results, a qualitative observation was that many ^64^Cu-ATSM-incubated cells, both CHO wild type and xrs5, had what appeared to be large and intense foci, along with many regular foci. These are thought to be clustered γH2AX foci [31], potentially resulting from an Auger cascade. In CHO wild-type cells with 0.8 Bq/cell uptake, ∼10 foci were produced and ∼80% of cells were dead (see Figs 2A and 1C). Based on previous research with CHO cells, 1 Gy of X-rays produced ∼30 foci and killed 10–20% of cells, and 1 Gy of carbon ions with LET 70 keV/μm produced ∼20 foci and killed 50% of cells [32]. According to this, ^64^Cu-ATSM exposures killed cells efficiently, with a smaller number of γH2AX foci formation (like high-LET radiation). This efficient cell killing may suggest that ^64^Cu-ATSM-induced γH2AX foci are more like high-LET radiation–induced clustered γH2AX foci. Therefore, these quantitative and qualitative observations of γH2AX foci after ^64^Cu-ATSM exposures provide further support to our hypothesis.

Finally, ^64^Cu-ATSM induced severe chromosomal aberrations in xrs5 cells, suggesting high-LET radiation exposure. Chromosomal aberrations, especially chromatid-type aberrations, were increased with ^64^Cu-ATSM treatment in both of the cell types. Radiosensitive xrs5 cells showed a number of massive multi-chromosome-associated chromosomal aberrations in high-activity ^64^Cu exposure (Fig. 3). Chromatid-type aberrations can be formed from (i) non-double-strand breaks in G1 phase and converted into double-strand breaks during replication collapses and (ii) post-replication double-strand breaks. High-LET radiation often produces severe DNA damage in exposed cells [32]. It is assumed that the massive damage in G1 phase tends to stop or slow down the cell cycle. This may be a reason why S/G2 damage–originated chromatid-type aberrations were dominant in both CHO wild-type and xrs5 cells after ^64^Cu-ATSM treatment.

^125^I is the well-studied Auger electron–emitting radionuclide often used as a thymidine analog in the form of ^125^Iodine-5-iodo-2′-deoxyuridine (^125^IUdR). ^125^IUdR is incorporated into DNA and efficiently produces DNA damage leading to high lethality. A level of 90% cell killing can be achieved with <0.01 Bq/cell [33]. ^64^Cu-ATSM produced 90% cell killing with 1 Bq/cell (Fig. 2B). This 100-times lower efficiency for cell killing can be explained by the cellular localization of ^64^Cu. Previous reports have shown that ^64^Cu distributes to cellular organelles, including nuclei, mitochondria and S2 fractions [11]. Therefore, the lower efficiency for cell killing can be explained with the lower accumulation of ^64^Cu in or near DNA compared with ^125^IUdR.

Auger electrons from ^64^Cu decay are considered as high-LET radiation [10]. When ^64^Cu reaches cellular nuclei, cells will be killed by Auger electron exposure but not by other types of radiation. We showed many reasons why ^64^Cu-ATSM-induced cell death is due to high-LET Auger electron exposure. Based on our results, future experimental and clinical research should focus on the application of ^64^Cu-ATSM high-LET theranostic therapy in the management of radioresistant tumors.

## FUNDING

This study is partially supported by the National Institute of Radiological Sciences President's Special Grant (YF), the International Open Laboratory (AF), the Colorado Clinical and Translational Sciences Institute (TAK), the Dr Akiko Ueno Radiobiology Research Fund (TAK), the Nuclear Regulatory Commission (NRC-38-10-951 for DM) and the Colorado State University Department of Environmental Radiological Health Sciences. Funding to pay the Open Access publication charges for this article was provided by Dr Akiko Ueno Radiobiology Research Fund.
